# Short and Long-Term Effects of Telaprevir on Kidney Function in Patients with Hepatitis C Virus Infection: A Retrospective Cohort Study

**DOI:** 10.1371/journal.pone.0124139

**Published:** 2015-04-29

**Authors:** Meghan E. Sise, Elke S. Backman, Julia B. Wenger, Brian R. Wood, Paul E. Sax, Raymond T. Chung, Ravi Thadhani, Arthur Y. Kim

**Affiliations:** 1 Division of Nephrology, Department of Medicine, Massachusetts General Hospital, Harvard University, Boston, MA, United States of America; 2 Department of Pharmacy, Massachusetts General Hospital, Harvard University, Boston, MA, United States of America; 3 Division of Allergy and Infectious Diseases, University of Washington, Seattle, WA, United States of America; 4 Division of Infectious Diseases, Brigham and Women’s Hospital, and Harvard Medical School, Boston, MA, United States of America; 5 Gastrointestinal Unit, Department of Medicine, Massachusetts General Hospital, Harvard University, Boston, MA, United States of America; 6 Division of Infectious Diseases, Department of Medicine, Massachusetts General Hospital, Harvard University, Boston, MA, United States of America; Kaohsiung Medical University Hospital, Kaohsiung Medical University, TAIWAN

## Abstract

**Background:**

Recent reports suggest that telaprevir, a protease inhibitor used to treat hepatitis C infection, is associated with decline in kidney function during therapy, particularly in patients with baseline renal impairment.

**Methods:**

Patients treated with telaprevir in a single healthcare network were retrospectively reviewed. Kidney function was determined at baseline, during therapy, and twelve weeks and twelve months after telaprevir discontinuation. Significant creatinine rise during therapy was defined as an increase in serum creatinine ≥ 0.3mg/dL from baseline during treatment with telaprevir.

**Results:**

Between July 2011 to January 2013,seventy-eight patients began treatment. The majority completed the prescribed twelve weeks of telaprevir therapy; 32% discontinued due to side effects. The average rise in serum creatinine during therapy was 0.22mg/dL (standard deviation 0.22mg/dL). Thirty-one percent experienced a significant creatinine rise during therapy. Decline in estimated glomerular filtration rate (eGFR) was lower in those with baseline eGFR < 90 mL/min/1.73m^2^ compared to the group with baseline eGFR ≥ 90 mL/min/1.73m^2^ (12 *vs*. 18 mL/min/1.73m^2^, P = 0.047). Serum creatinine fully normalized by twelve weeks after cessation of telaprevir in 83% of patients, however experiencing a significant creatinine rise during telaprevir use was associated with a 6.6mL/min/1.73m^2^ decrease in estimated glomerular filtration rate at twelve months in an adjusted model.

**Conclusions:**

Decline in kidney function during therapy with telaprevir is common and is not associated with baseline eGFR < 90mL/min/1.73m^2^ as previously reported.

## Introduction

Telaprevir is a first-generation protease inhibitor that was approved by the US FDA in 2011 to treat hepatitis C virus (HCV) infection. When used in combination with pegylated interferon (PegIFN) and ribavirin (RBV), cure rates for genotype 1 infection improved substantially compared to treatment with PegIFN and RBV alone[1–5]. Discontinuation due to hematologic, gastrointestinal and dermatologic side effects is common[6, 7], yet renal impairment was not initially noted in the adverse events profile seen in early trials[3, 4]. Recent case reports highlight that telaprevir may be associated with acute kidney injury [8–11]. Large series have suggested up to 25% of patients may have an increase in serum creatinine during therapy[12], with up to 6.6%-17% experiencing a decrease in estimated glomerular filtration rate (eGFR) below 60mL/min/1.73m^2^[13, 14]. Changes in kidney function are not typically noted in patients treated with PegIFN and RBV alone[13, 14]. It has been suggested that baseline renal impairment is associated with increase risk of decline in renal function during telaprevir use[13]. However, there is controversy in the field because previous studies used a study endpoint of decline in eGFR below 60mL/min/1.73m^2^ rather than determining absolute change in serum creatinine or eGFR. Furthermore, all previous studies only examined short-term follow-up of kidney function following cessation of therapy. No prior studies have examined long-term kidney function to determine if there is a lasting effect of telaprevir on kidney function after discontinuation of this medication.

The purpose of this study was to determine the change in serum creatinine and eGFR during therapy with telaprevir, identify if baseline eGFR is associated with changes in serum creatinine during therapy, and assess the reversibility of these changes after discontinuation of telaprevir. We also sought to determine if decline in eGFR during therapy with telaprevir affected kidney function at one year of follow-up.

## Subjects and Methods

### Subjects

Patients were who began telaprevir-based triple therapy for HCV infection at our institution between July 2011 and January 2013 were included if they had at least one serum creatinine measured prior to initiation of telaprevir-based therapy and one repeat serum creatinine measured while taking telaprevir. Their electronic medical records were reviewed. Patients were eligible for telaprevir treatment if they were age at least 18 years old, had chronic hepatitis C infection (all had genotype 1 infection) and had a baseline eGFR greater than 50mL/min/1.73m^2^. The Chronic Kidney Disease Epidemiology Collaboration (CKD-EPI) formula was used to determine eGFR because this equation more accurately characterizes kidney function in patients with normal or near-normal kidney function[15]. Baseline eGFR was calculated based on the serum creatinine measurement prior to treatment initiation. All patients were included in the analysis regardless of whether or not they completed therapy.

**Fig 1:**
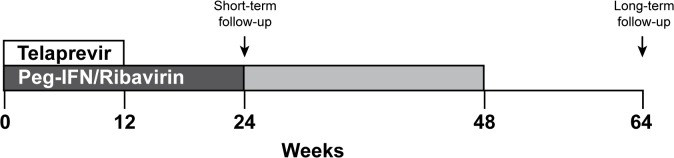
Timeline of Triple Therapy, Short and Long-term Follow-up. Patients were prescribed triple therapy (telaprevir, Peg-IFN, and ribavirin) for twelve weeks. Based on clinical characteristics and at the providers discretion patients then continue Peg-IFN and ribavirin for a total of 24–48 weeks. Short-term follow-up was obtained 12 weeks after telaprevir was discontinued (at week 24). Long-term follow-up was obtained 12 months after telaprevir was discontinued (at week 62).

At the discretion of the treating physician, triple therapy with telaprevir, PegIFN and RBV was prescribed for 12 weeks, followed by dual therapy with PegIFN and RBV for either 24 or 48 weeks total (Fig 1). Serum creatinine values were obtained during therapy with telaprevir and the maximum values used to determine the presence of significant creatinine rise during telaprevir use. A significant creatinine rise was defined as a ≥ 0.3mg/dL increase from baseline. This cutoff was chosen as it is the same magnitude of rise that has been shown to have significant effect on chronic kidney disease development and progression in the setting of acute kidney injury[16]. Patients were considered to have cirrhosis if it was demonstrated on liver biopsy or if the treating physician determined it to be likely based on clinical findings, imaging, and fibrosis score. Sustained viral response (SVR), or cure of hepatitis C, was defined as undetectable HCV RNA 12 weeks after therapy[17]. Short-term kidney function recovery was determined based on serum creatinine between 4–12 weeks after discontinuation of telaprevir, at this time point,patients were still on PegIFN and RBV unless they had prematurely discontinued therapy due to side effects. Normalization of kidney function was defined as return of eGFR to at least 90% of prior baseline within 12 weeks after discontinuing telaprevir. Long-term kidney function recovery was determined based on serum creatinine between twelve months after discontinuation of telaprevir. See Fig 1 for a schematic representation of treatment course and follow-up time-points.

### Statistical Analysis

Baseline characteristics of all patients with at least one baseline and one serum creatinine measurement during therapy with telaprevir were described using mean ± standard deviation or percent. Race was determined by self-report. Baseline and follow-up eGFR were compared using paired samples t-tests. Univariate linear regression models were used to determine baseline factors associated with change in eGFR during therapy. Multivariable linear regression model predicting change in eGFR during and after therapy with telaprevir included adjustment for clinically relevant baseline characteristics including age, gender, baseline eGFR, diabetes mellitus, cirrhosis, HIV co-infection and any other variables that were significant in univariate models. A logistic regression model was used to determine baseline factors associated with significant creatinine rise during telaprevir therapy; odds ratios and 95% confidence intervals were used to summarize the results of this model. Because previous studies have suggested that baseline renal impairment, defined as eGFR less than 90mL/min/1.73m^2^ is associated with increased risk of decline in renal function with telaprevir, we compared changes in kidney function in patients with baseline eGFR < 90mL/min/1.73m^2^ compared to those with eGFR ≥ 90mL/min/1.73m^2^[13].

Signs and symptoms that led to discontinuation of telaprevir in the subset of patients who terminated therapy early were reviewed. Reasons for discontinuation were compared in patients with significant creatinine rise during telaprevir treatment and those without. All analyses were performed with Stata version 13.1. For all comparisons, a two sided P value of <0.05 was considered to indicate statistical significance.

The IRB at Partners Healthcare System approved this study, protocol 2012P000970. The need for informed consent was waived because the data were collected retrospectively and analyzed anonymously. A limited de-identified dataset release was approved by the Partners IRB and is available (S1 Table).

## Results

Seventy-eight patients were treated with telaprevir and had at least one serum creatinine measured during therapy. Baseline characteristics are summarized in Table 1. The population was predominantly white, two-thirds were male, and the mean age was 55 (SD ± 8.7) years. The average baseline serum creatinine was 0.87mg/dL ±0.21 mg/dL corresponding to a mean baseline eGFR 91mL/min/1.73m^2^ ±18mL/min/ 1.73m^2^. The majority (56% percent) of the cohort had baseline eGFR ≥90mL/min/1.73m^2^, 39% percent had a baseline eGFR 60-90mL/min/1.73 m^2^, and 5% had a GFR between 50-60mL/min/1.73m^2^.

**Table 1:** Baseline demographics and clinical characteristics of patients treated with telaprevir (n = 78).

	Mean ± SD or %
Age (years)	55 ± 9
Male (%)	67%
Race (%)	
White, non-Hispanic	82%
Hispanic	10%
Asian	4%
Black	3%
Other/Unknown Race	1%
Cirrhosis (%)	55%
HIV (%)	13%
Diabetic (%)	17%
Body Mass Index (kg/m2)	28.4 ± 4.7
Smoking History	
Current smoker	23%
Former smoker	38%
Never smoked	39%
Alcohol Use (%)	
Current social use	22%
Prior social use	33%
Prior abuse	32%
None	12%
Prior intravenous drug use (%)	47%
Previously treated with Ribavirin and Interferon (%)	67%
Baseline Serum Creatinine (mg/dl)	0.87 ± 0.2
Baseline eGFR (mL/min/1.73m2)	90.8 ± 17.7
eGFR ≥ 90	56%
eGFR 60–89	39%
eGFR < 60	5%
Completed 12-week telaprevir course (%)	65%
Telaprevir discontinued early (%)	35%
Mean duration of telaprevir (weeks)	7.0 ± 0.7
Baseline HCV Viral Load (median copies/mL)	2,900,000
Percent achieving SVR	60%

^HIV = Human Immunodeficiency Virus, eGFR = estimated glomerular filtration rate, HCV = hepatitis C virus, SVR = sustained viral response^

**Fig 2:**
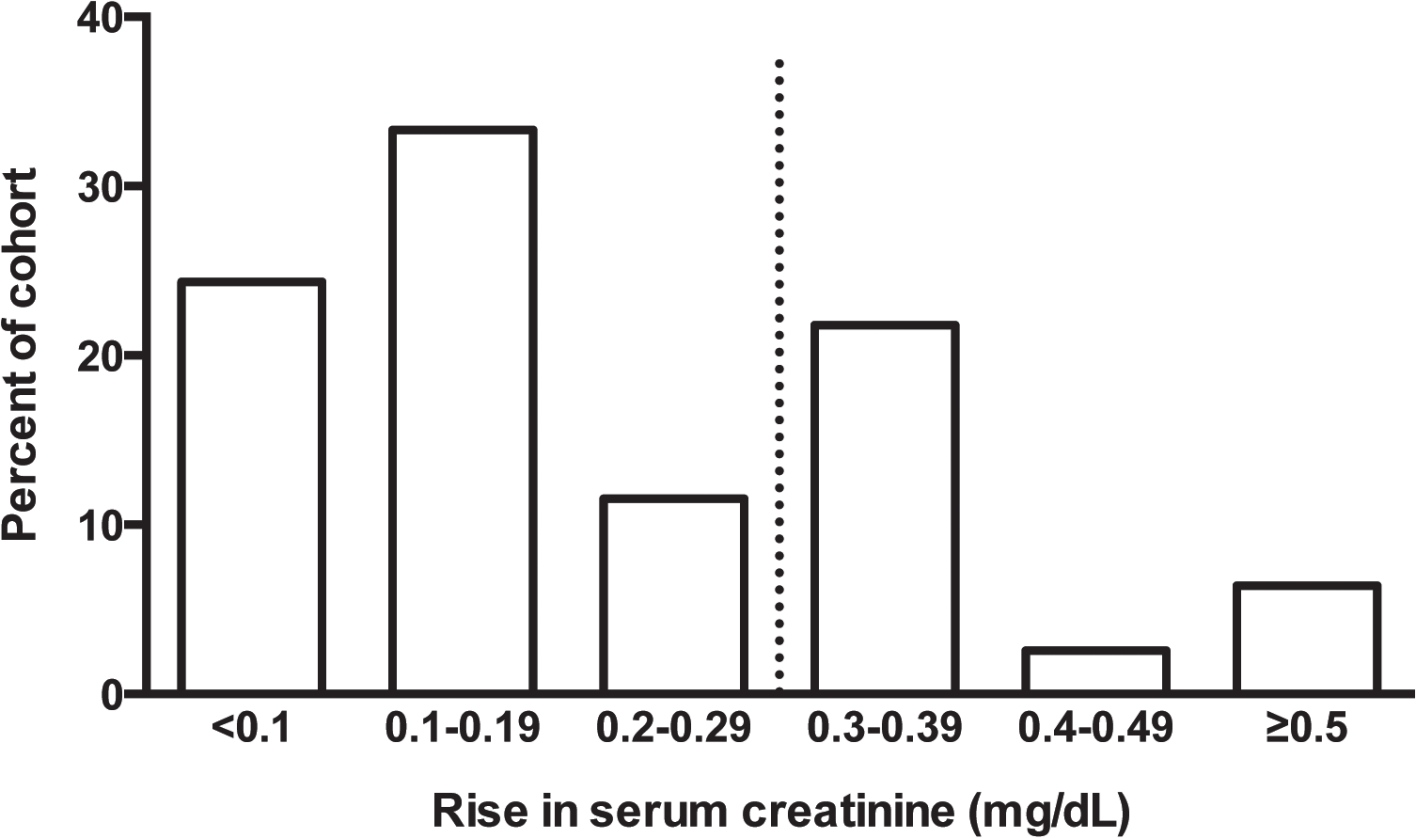
Peak rise in serum creatinine during telaprevir therapy. Bar graphs represent the numbers of patients who experienced a maximum rise in serum creatinine in the above ranges. The majority (55%) of patients experienced a small (< 0.2mg/dl) rise in creatinine during telaprevir therapy. However, thirty-one percent developed significant creatinine rise ≥ 0.3mg/dl, shown by a dashed vertical line.

Elevation of serum creatinine during therapy with teleprevir was common (Fig 2). The serum creatinine was increased by an average of 0.22 mg/dL (±0.22mg/dL, P<0.001), and the average decline in eGFR was 15.7 mL/min/1.73m^2^ (±13.4mL/min/1.73m^2^, P<0.001) when exposed to telaprevir. Twenty-three percent of the cohort had an eGFR that dropped below 60mL/min/1.73m^2^ during telaprevir therapy, compared to 5% with eGFR less that 60mL/min/1.73m^2^ at baseline. Overall, thirty-one percent of the cohort met the definition of significant creatinine rise (0.3mg/dL) during the period they were treated with telaprevir. Elevation of serum creatinine ≥ 0.5mg/dL occurred in 6% of the cohort (Fig 2). Hepatitis C cure, or SVR, was achieved by 60% of the cohort. There was no effect of baseline eGFR on the likelihood of achieving SVR (61% for eGFR < 90 mL/min/1.73m^2^ vs. 59% eGFR ≥ 90 mL/min/1.73m^2^ P = 0.8). Although not statistically significant, there was a trend toward increased likelihood of achieving SVR in those who experienced a significant creatinine rise during therapy compared to those who did not (70% vs. 55%, P = 0.2).

### Analysis based on baseline eGFR

**Fig 3:**
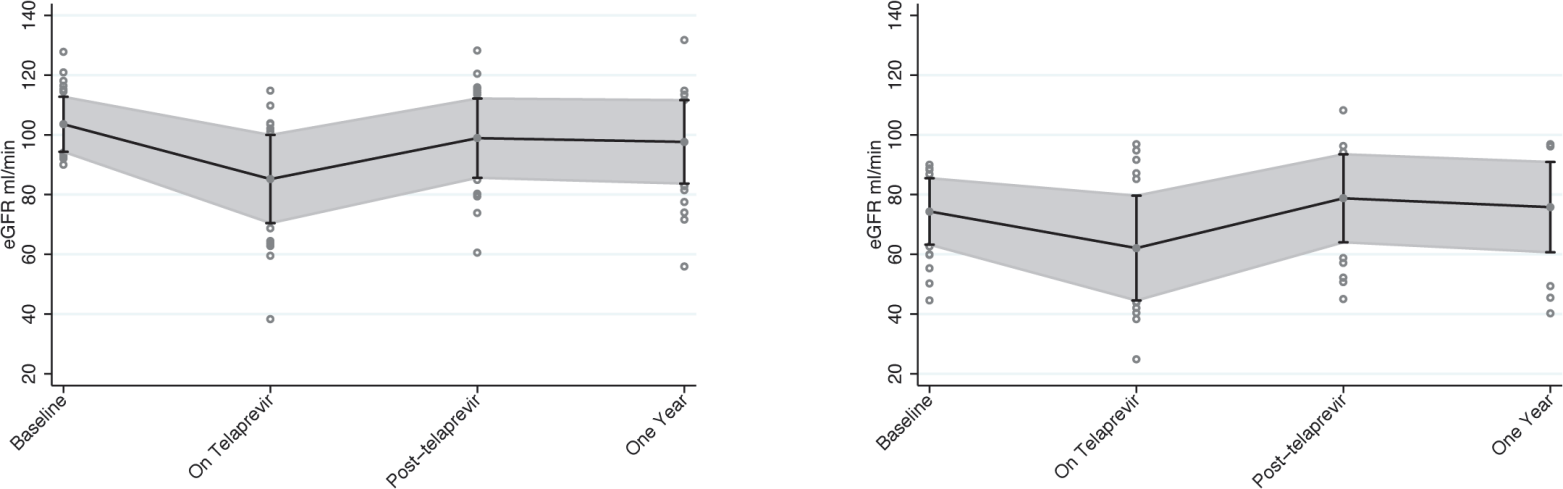
Average estimated glomerular filtration rate in patients during and after telaprevir therapy, by baseline kidney function. 3a. Baseline eGFR ≥ 90 mL/min/1.73m^2^ 3b. Baseline eGFR < 90 mL/min/1.73m^2^. Mean eGFR (solid gray circle) and one-standard deviation error bars are shown with shading at baseline, on treatment (nadir), 12 weeks after finishing telaprevir, while still on PegIFN and RBV, and then one year after completing telaprevir, off all treatment. P < 0.01 for changes between baseline eGFR and eGFR while on telaprevir in both groups. There is no signficant difference between baseline eGFR and eGFR 12 weeks or one year post telaprevir. Fig 3a shows patients with baseline eGFR ≥ 90mL/min/1.73m^2^. (N = 44). 3b.) Includes patients with baseline eGFR < 90mL/min/1.73m^2^ (N = 34). Values that fell outside of one standard deviation are shown with hollow gray circles. eGFR = estimated glomerular filtration rate.

When patients with eGFR < 90mL/min/1.73m^2^ at baseline were compared to those with eGFR ≥ 90 mL/min/1.73m^2^ at baseline the average decline in eGFR was, in fact, slightly lower in those with eGFR < 90 mL/min/1.73m^2^ compared to those with eGFR ≥ 90mL/min/1.73m^2^, 12 *vs*. 18mL/min/1.73m^2^, respectively, P = 0.047. Changes in eGFR during and after treatment stratified by baseline eGFR are shown in Fig 3A and 3B. When using a cutoff eGFR of 60mL/min/1.73m^2^ to define “renal impairment” as prior studies have done,[13] then unsurprisingly, the likelihood of this outcome was increased in the group with eGFR < 90 mL/min/1.73m^2^ (40% vs. 4.5%, P < 0.001). Baseline eGFR did not have an effect on the odds of significant creatinine rise during treatment in a multivariable logistic model (Table 2). There were no baseline demographic or clinical characteristics that were associated with the development of significant creatinine rise during telaprevir therapy.

**Table 2:** Univariate and multivariable logistic regression model predicting development of significant creatinine rise during therapy with telaprevir.

	Univariate Model		Multivariable Model	
Baseline predictors of significant creatinine rise during therapy	Odds Ratio (95% CI)	P value	Odds Ratio (95% CI)	P value
Age, per 10 years	1.75 (0.89, 3.44)	0.1	2.34 (0.94, 5.78)	0.066
Sex (male versus female)	1.30 (0.46, 3.74)	0.6	1.22 (0.37, 3.98)	0.74
Diabetes (versus non-diabetic)	2.2 (0.66, 7.57)	0.19	2.40 (0.65, 8.93)	0.19
Cirrhosis (versus non-cirrhotic)	1.2 (0.46, 3.19)	0.7	0.71 (0.21, 2.38)	0.58
HIV coinfection	1.60 (0.41, 6.29)	0.58	3.33 (0.66, 16.70)	0.14
Baseline eGFR, per 10ml/min increase	0.93 (0.70, 1.22)	0.92	1.09 (0.77, 1.56)	0.6

^Abbreviations: HIV = human immunodeficiency virus, eGFR = estimated glomerular filtration rate^

### Short-term recovery and one year follow-up

Short-term follow-up 12 weeks after telaprevir was discontinued was available on 71 patients (91% of the cohort). The majority (68%) was still on PegIFN and RBV at this time-point, however 32% had discontinued triple therapy due to side effects and the 12-week follow-up value is off all treatment in those cases. Creatinine measurements after telaprevir discontinuation returned to within 90% of baseline levels at 12-week follow-up in 83% of patients; on average there was no significant difference in baseline eGFR and eGFR post telaprevir discontinuation when compared in a paired analysis (91.2 vs. 90.6 mL/min/1.77m2, P = 0.67). There was a trend toward increased likelihood of normalization of serum creatinine to baseline in those patients who remained on PegIFN and RBV compared to those who discontinued all triple therapy, (88% vs. 70% returned to within 90% of their baseline eGFR, P = 0.07).

Long-term follow-up, 12 months after telaprevir was discontinued, was available on sixty-four patients (82% of the cohort). There were no differences in baseline kidney function or rate of significant creatinine rise during telaprevir therapy in patients lost to follow-up (P = 0.87 and P = 0.14, respectively). Serum creatinine was available at a median of 444 days after discontinuing telaprevir (IQR 392–505 days). In the univariate model, baseline eGFR, presence of diabetes, cirrhosis and significant creatinine rise during telaprevir therapy were associated with decline in eGFR at 12-month follow-up. In the adjusted model, patients who experienced significant creatinine rise during telaprevir therapy had a 6.6 mL/min/1.73m^2^ lower eGFR at 12-month follow-up (P = 0.03, Table 3).

**Table 3:** Univariate and multivariable linear regression model predicting change in eGFR over one year follow-up.

	Univariate Model		Multivariable Model	
Predictors of change in eGFR at one year (ml/min/1.73m2 difference from baseline)	β Coefficient (95% CI)	P value	β Coefficient (95% CI)	P value
Age, per 10 years	0.40 (-3.33, 4.20)	0.82	-0.64 (-5.12, 3.84)	0.77
Sex (male versus female)	-0.86 (-6.97,5.20)	0.78	2.5 (-3.48, 8.53)	0.4
Diabetes (versus non-diabetic)	-10.13 (-17.47, -2.79)	0.008	-6.09 (-13.45, 1.27)	0.1
Cirrhosis (versus non-cirrhotic)	-5.67 (-11.43, -0.99)	0.05	-4.21 (-10.58, 2.15)	0.19
HIV coinfection	4.46 (-3.90, 12.83)	0.29	5.19 (-3.42, 13.82)	0.23
Baseline eGFR, per 10ml/min increase	-2.19 (-3.72, -0.66)	0.006	-1.18 (-3.70, 0.15)	0.071
Significant creatinine rise during telaprevir	-6.65 (-29.74, -3.25)	0.02	-6.31 (-12.08, -0.53)	0.03

^Abbreviations: HIV = human immunodeficiency virus, eGFR = estimated glomerular filtration rate^

### Side effects and treatment discontinuation

Thirty-two percent of the cohort discontinued telaprevir prior to twelve weeks of therapy due to side effects. Decline in eGFR on telaprevir did not differ between those who did and did not discontinue telaprevir therapy; there was on average a 17.9 *vs*. 14.6mL/min/1.73m^2^ decline, respectively (P = 0.3). Although 44% of patients who discontinued telaprevir experienced significant creatinine rise during therapy, acute kidney injury was the primary reason for discontinuation in only one case. There were differences between the signs and symptoms that led to discontinuation in patients who met the definition of significant creatinine rise compared to those who did not, however the numbers were small and the majority of these differences did not reach statistical significance (Table 4). In the lone case where acute kidney injury contributed to the discontinuation of telaprevir, a male patient’s serum creatinine rose from a baseline of 1.5mg/dL to 2.69mg/dL (eGFR 50 to 25mL/min/1.73m^2^) three weeks after starting telaprevir in the context of severe fatigue and decreased oral intake with ongoing diuretic and angiotensin converting enzyme inhibitor use. The systolic blood pressure was 80mmHg at the time of presentation to the emergency room. There was no evidence of hemolysis. Urinalysis was negative for red blood cells, white blood cells and protein and the etiology of acute kidney injury was presumed to be acute tubular necrosis due to hypotension. Serum creatinine returned to baseline 1.49mg/dL within four weeks.

**Table 4:** Symptoms contributing to the discontinuation of telaprevir (n = 25).

Signs or Symptoms	# of patients with creatinine rise	# of patients without creatinine rise
	Total N = 11	Total N = 14
Symptomatic anemia, fatigue or dyspnea	5	6
Viral breakthrough	1	3
Gastrointestinal symptoms ^ϕ^	3	0
Rash	1	1
Encephalopathy	0	2
Hallucinations	0	2
Thrombocytopenia	0	1
Acute kidney injury	1	0
Other*	0	2

^Twenty-five patients discontinued telaprevir therapy. Numbers may add up to more than total N if combinations of multiple symptoms led to discontinuation.^

^**ϕ -** Significant at the level of P < 0.05^

^*Other reasons for discontinuation that each affected one patient included fever and noncomplicance.^

## Discussion

This study confirms that a large proportion of patients experience a significant decline in eGFR during therapy with telaprevir; however in contrast to previous studies, baseline renal impairment was not a risk factor for significant creatinine rise during telaprevir use. Thirty-one percent of patients had a creatinine rise ≥ 0.3mg/dL during therapy with telaprevir. Twenty-three percent of our cohort experienced a decline in eGFR below 60mL/min/1.73m^2^ during treatment, a greater percentage than seen in prior cohorts[13]. This is likely because nearly half of our cohort had an eGFR less than 90mL/min/1.73m^2^ prior to starting telaprevir, reflecting the high rates of comorbid conditions existing in this patient population, including cirrhosis, HIV infection and diabetes, which are all associated with chronic kidney disease[18–20].

Our results confirm findings of Karino *et al*. who reported that patients treated with telaprevir-based triple therapy experienced on average a 15-20mL/min/1.73m^2^ decline in eGFR that persisted throughout the duration of treatment in telaprevir and Virlogeux *et al*. who found that 40% of their cohort had a maximal eGFR decrease more than 20 mL/min/1.73m^2^ [21, 22]. Loustad-Ratti *et al*. studied a cohort of 36 patients receiving telaprevir with frequent serum creatinine measurements and showed that eGFR declined by 15mL/min/1.73m^2^ within four weeks of starting telaprevir based triple therapy, remained at this level throughout the duration of telaprevir exposure, and normalized 4 weeks after discontinuation[14]. Importantly, our findings contradict the conclusions of Mauss *et al*., who determined that baseline eGFR < 90 mL/min/1.73m^2^ predicts decline in renal function during telaprevir use. This group reported on 575 patients treated with telaprevir and determined that patients with “baseline renal impairment” (eGFR 60-90mL/min/1.73m^2^) were more likely to experience a decline in eGFR less than 60mL/min/1.73m^2^ at week 12 of therapy. While this is true, using this outcome does not adequately describe what occurs in patients treated with telaprevir. In fact, we found that the absolute change in kidney function was actually less in those with eGFR < 90mL/min/1.73m^2^ compared to those with eGFR ≥ 90mL/min/1.73m^2^. Furthermore, previous studies that designated baseline eGFR 60-90mL/min/1.73m^2^ as “impaired renal function” are misleading, as in the absence of other signs of kidney damage including proteinuria or hematuria, this eGFR range is considered normal[13].

We confirm prior studies showing that eGFR normalized after cessation of telaprevir[14, 21]. In the majority of cases, patients were still on PegIFN and RBV. However, this is the first study that evaluated kidney function with an extended period of follow-up, over twelve months after cessation of telaprevir. While it is reassuring that most patients’ kidney function recovered to their baseline after medication discontinuation, it is concerning that long term loss of eGFR after therapy discontinuation was greater in those who experienced significant creatinine rise during therapy with telaprevir, even after adjustment for other variables that predict decline in eGFR over time. The clinical significance of this small decline in eGFR remains uncertain.

Previous studies have suggested that the change in serum creatinine associated with telaprevir use supports *in vitro* data suggesting that telaprevir may impair creatinine secretion and that the rise in serum creatinine may not be a sign of renal failure[13, 23]. However, neither this nor previous studies have been designed to answer this question as no patient underwent simultaneous iohexol clearance testing. Furthermore, the lack of systematic evaluation of patients with urinalysis, microscopy, or proteinuria quantification during therapy in this or other studies limits the ability to exclude nephrotoxic tubular damage. There are currently no reports in the literature of patients with acute kidney injury during telaprevir use that underwent kidney biopsy. Thus, nephrotoxic injury that fully or partially resolves is a possible explanation for the pattern of change in eGFR during and after treatment, supported by the fact that there is an appreciable change in long term kidney function in patients who experienced a significant creatinine rise while taking telaprevir.

There are significant limitations in our dataset. First, this is a small series of patients of predominantly Caucasian background; larger series may have identified a number of patients with persistent renal impairment after telaprevir use. Additionally, these results may not apply to individuals of different ethnic backgrounds. The use of a single serum creatinine measurement to estimate baseline eGFR may have led to misclassification of kidney function groups. Even though this study provides the only available extended follow-up eGFR assessment, a year is still considered only a modest length of follow-up in terms of the likelihood of detecting progression of kidney disease. Another limitation of this study is that we cannot exclude that the rise in serum creatinine is due to the effect of PegIFN and RBV since these medications were always co-administered with telaprevir, however, the fact that serum creatinine normalized while still 68% of the cohort was still on PegIFN and RBV suggests that telaprevir was the main cause of elevated creatinine. It is well established that a creatinine rise of ≥ 0.3mg/dL is a risk factor for developing chronic kidney disease and progression to end stage renal disease[24–27]. Yet, whether the small change in eGFR at one year in this series of patients who experienced significant rise in creatinine translates into patient-important outcomes would require larger series with longer follow-up.

HCV infection itself is an established risk factor for chronic kidney disease progression[28–30]. Data suggest that HCV itself may be a reversible risk factor for progression to end-stage renal disease if adequately treated[31, 32]. The findings in this study are particularly useful for those in the developing world still using telaprevir. Newer agents that have improved safety and tolerability profiles will supplant telaprevir in resourced settings. However, it will be important to assess both the short term and long term renal outcomes in patients with HCV who undergo treatment with novel agents in order to understand their effects on renal function. This study highlights the importance of determining both the absolute change in eGFR in addition to determining the percent with eGFR declining below 60mL/min/1.73m^2^ to assess the effect of new medications on kidney function to avoid falsely concluding that eGFR < 90mL/min/1.73m^2^ is a risk factor for renal impairment on therapy.

## Supporting Information

S1 Table Limited patient dataset. Age is rounded to the nearest 10 years to protect patient privacy. Abbreviations: HIV = human immunodeficiency virus, eGFR = estimated glomerular filtration rate.
(XLS)
